# Study of purchasing behavior evolution of work-safety-service based on hierarchical mixed supervision

**DOI:** 10.3389/fpsyg.2022.991539

**Published:** 2022-11-30

**Authors:** Zhong Jingjing, Li Wei

**Affiliations:** School of Economics and Management, Changzhou Institute of Technology, Changzhou, Jiangsu, China

**Keywords:** work-safety-service, ex ante supervision, ex post supervision, hierarchical mixed supervision, evolutionary game

## Abstract

This paper explores the evolutionary rules of work-safety-service purchasing strategies of enterprises under hierarchical mixed supervision. Considering the influence of the central government’s inspection on local governments, an evolutionary game model is built which depicts the mutual interactions of work-safety-service purchasing strategies from enterprises and supervision strategies from local governments. The existence and stability of system equilibrium points are analyzed, and the influence of different parameters’ variation on the evolutionary results is demonstrated through numerical simulation. It is found that different ranges of parameters affect the number and stability of equilibrium points and the evolutionary trend. The system converges to two different patterns. In the first pattern, local governments choose to supervise enterprises strictly and enterprises choose to purchase work-safety service, which is a desired pattern. In the second pattern, local governments choose to supervise enterprises loosely and enterprises choose not to purchase work-safety service, which is an undesired pattern. When it has five equilibrium points, it is feasible to make the system converge to the desired pattern through modifying relative parameters, avoiding the undesired pattern. The system is more likely to converge to the desired pattern with the higher initial ratio of local governments opting for strict enterprise supervision; the system is more likely to converge to the desired pattern when the central government exerts a stricter inspection on local governments; the system is more likely to converge to the desired pattern when local governments exert stricter ex ante and ex post safety supervision on enterprises. The system is more likely to converge to the undesired pattern when the ex ante supervision costs of local governments get higher. Subsidies from local governments for the purchase of work-safety service barely affect the evolutionary trend of the system.

## Introduction

Major accidents with overwhelming damage happen from time to time, albeit great enforcement of Work safety Act and strong resolution of Central People’s Government to improve work safety condition in enterprises. Taking Hushan gold mine blast accident of Five Color Dragon Investment Co., LTD as an example, a *post hoc* investigation reveals several safety supervision issues. The enterprise does not have professional blasting technology and safety experts at site for safety management. Safety regulation and supervision from the local government does not play due role (Duan, 2021). It is not difficult to find out that the rapid development of enterprises requires more professional work safety service, while the traditional way of government-oriented work-safety service can no longer meet the diversified requirements of enterprises, thus arising the market-oriented-service way characterized with “enterprises be responsible, governments supervise and service organizations participate” (Dai, 2022). In China, the emergence and development of the work-safety service market are consistent with the government role transition from regulatory agency to service provider (Wang et al., 2018; Han and Bae, 2021).

Based on theory of labor division and circuitous production, work safety service can be regarded as a nonphysical material inserted between the initial raw material and the end products, which cannot be ignored in the value chain in enterprises. The formation of work-safety-service market is a dynamic process with safety and health issues in enterprises becoming more and more complicated (Legg et al., 2015). When safety manager in enterprises aren’t able to deal with specific issues, professionals from work-safety organizations are needed. When there is demand and supply of work safety service, work-safety-service market is gradually emerging and developing (Zhong et al., 2015). Unlike general commodities in the market, value of work safety service is usually hard to manifest immediately, since accidents happen in enterprises with probability by chance. Enterprises only engage in purchase of work safety service when there is a strong incentive for them to do so; such incentives can include internal safety management needs or external supervision constraint from governments (Bragatto et al., 2015). Notably, demand of work-safety-service in market is inadequate because enterprises are not proactive enough to purchase work safety service. In these circumstances, how to urge enterprises to purchase work safety service is of great significance not only to enterprises but also to work-safety-service market.

It usually costs an enterprise a large amount of expenditure to improve its work safety condition, but the improvement is not easy to manifest. So enterprises are not proactive enough to invest in work safety management (Faure and Tilindyte, 2010; Li et al., 2022). Speaking of which, the government has a responsibility to promote purchase of work safety service which helps improve work safety condition in enterprises and protect workers. Local governmental supervision includes ex ante (incentive) supervision measures and ex post (restrictive) supervision measures. Ex ante (incentive) supervision measures refer to such policies as economic subsidies, tax incentive, and financial support, etc. Ex post (restrictive) supervision measures include accidents investigation, emergency rescue, accidents treatment, economical punishment, etc. (Zhou et al., 2020). Andersen et al. (2019) made an exploratory discussion on governmental regulatory modes, desired results and interrelationship between them (Andersen et al., 2019). He believed that the mandatory regulation has little positive effect, which makes governments resort to encouraging enterprises to reinforce self-management on work safety and increase work-safety investment (Vasin et al., 2017). The research of Umar et al. (2018) and Zhou et al. (2020) showed that appropriate punishments can increase the investment of enterprises in work safety management to a certain extent, but excessive punishments would make enterprises overwhelmed (Umar et al., 2018; Mei et al., 2020). Wang et al. (2018) and Merve (2019) claimed that the best way to deal with external market failure caused by accidents in enterprises is to make full use of the government’s social regulation, such as ex ante supervision and incentive, which is more effective than ex post measures (Wang et al., 2018; Merve, 2019). Chan et al. (2010, 2011) studied the Pay for Safety Scheme (PFSS) led by government through questionnaire and interview. He found that the economic incentives from governments could effectively alleviate funding shortage in enterprises and promote investment of work-safety management in construction enterprises (Chan et al., 2010; Choi et al., 2011). However, Xu’s (2019) research revealed that for an enterprise, work-safety investment is not only depending on the ex ante supervision but also on the ex post punishments (Xu and Shi, 2019). Kolstad et al. (1990) and Schmitz (2000) made an quantitative analysis through a probability model, concluding that how ex ante and ex post supervision is organized and conducted, and with which impact on work-safety-service purchase in enterprises depended on many factors, such as information symmetry degree among governments, enterprises and the injured people, legal system integrity and negative externality caused by specific accidents in enterprises. Namely, in order to supervise enterprises effectively, it is necessary to analyze and decide based on specific situations when it comes to what kind of regulatory measures should be taken (Kolstad et al., 1990; Schmitz, 2000). Shen (2012) compared the effectiveness of ex ante and ex post supervision based on the system dynamics model. It is discovered in his research that ex post supervision efforts is positively associated with benefits in governments and enterprises; strengthening ex post supervision is beneficial to encourage enterprises to invest more in work safety management, thus improving the effect of work safety supervision and the overall social welfare (Shen, 2012). Hu and Liu (2008) pointed out in a game model of coal mine work safety supervision that the severe punishments after accidents could not curb the violations of coal mine enterprises at all, but might lead to more serious coal mine accidents instead. Therefore, he proposed that ex post supervision combined with ex ante economic incentives might make coal mine enterprises invest on work-safety management spontaneously (Hu and Liu, 2008).

It is implied in those literatures that work-safety-service purchase, as an important work safety investment, which accounts for a large amount of expenditure in enterprises, is influenced by both ex ante (incentive) supervision and ex post (restrictive) supervision from governments. It should be noted that local governments are the direct supervisor of enterprises and the direct executor of work safety policies as well; the central government is responsible for work safety policies making and implementation. The central government represents the interests of the whole public and it’s the major beneficiary of work safe policy implementation; local governments pay for the work safety supervision literally and enterprises pay for work safety policies implementation literally. It is very tricky that the major evaluation indicators and the promotion of local government officials depend on regional economic progress. Therefore, local governments do not always fully implement work-safe policies from the central government and evidence shows that Chinese local governments tend to cooperate with local enterprises to rapidly improve regional economic progress (Nie and Li, 2006; Li et al., 2009; Sun et al., 2020).

Existing literatures on work safety supervision from government are mainly focused on its impact on enterprises, rather than on specific promoting mechanisms to improve work safety condition in enterprises. Besides, analysis of how governmental supervision is organized and conducted, and with which impact on work-safety-service purchase in enterprises, is largely missing, presenting a significant gap. This paper aims to present a glimpse of how governmental work-safety supervision exerts influence on work-safety-service purchase in enterprises. In order to do so, we intend to build a game model that includes local governments and enterprises under ex ante and ex post supervision mechanism. We will use this model to investigate the strategic choices made by the participants in an evolutionary game theory framework. Considering that the central government plays a significant role in inspecting local governments and only has one strategy, we quantified its influence as several parameters rather than a participant. Evolutionary game theory is used to better explore the choices of local governments and enterprises. We seek to find an evolutionarily stable strategy (ESS) in the dynamic game process. The evolutionary paths of two participants can also be shown directly, and we can intuitively examine how each participant is affected by different factors in the evolutionary process. If a pure strategy of a participant is irrational, the probability of that pure strategy will be zero in any internal path of dynamic evolution. This implies that the ultimate ESS is a rational result. The evolutionary game is a dynamic adjustment process in a complex system. We use this model to study the conditions that determine the local government’s choice between supervising enterprises strictly and supervising enterprises loosely. We also study how local government and enterprises select strategies that could drive the dynamic game model to an ESS. We further analyze the factors that are crucial in encouraging enterprises’ work-safety-service purchase behavior and promoting work safety condition. We then discuss the practical feasibility of all of the equilibrium points and examine the evolutionary path of the participants through numerical simulation with the aid of MATALB. We finally make some suggestions for better encourage enterprises to purchase work-safety-service and foster work-safety-service market.

## Basic assumptions and model building

Evolutionary game theory transposes Darwinian mechanisms into a mathematical form by adopting a system model of evolutionary processes with three main components: population, game, and replicator dynamics. In order to reveal evolutionary rules on repeated game, we choose one participant from local governments and one participant from enterprises, and then we make them contest to discover their payoffs in a single game. The evolutionary equilibrium strategies between participants are always the results of learning and adjustment rather than one-shot game results; the game allows for errors and allows participants to learn from previous errors to reach stable state strategies (Jin and Zheng, 2022). Thus, evolutionary game theory is generally used to study the long-term stable strategy choice of participants. Evolutionary game theory emphasizes the results of interactive learning and strategy adjustment between participants. Although it is difficult to accurately predict the specific strategy of single participant (i.e., the local government and the enterprise in pairwise game), the evolutionary trend of two groups could be revealed as a process of participants’ learning and adjustments.

### Basic assumptions

*Hypothesis 1*: Local government groups and enterprise groups do not know each other’s exact strategy and payoffs in each single game. Two game players both have limited rationalities and they can only achieve equilibrium through continuous learning and optimization of their own strategies.

*Hypothesis 2*: Suppose the strategy space of local governments is {strict regulation, loose regulation}, denoted as { $A_{1},A_{2}$ }. Strict supervision strategy includes ex ante (incentive) supervision measures and ex post (restrictive) supervision measures. Ex ante (incentive) supervision measures refer to such policies as economic subsidies, tax incentive, and financial support, etc., which cost local government ( $C_{1}$ ); ex post (restrictive) supervision measures include accidents investigation, emergency rescue, accidents treatment, economical punishment, etc., which cost local government ( $C_{2}$ ). Notably, when the local governments choose to supervise enterprises loosely, supervision strategy only refers to ex post supervision measures with cost ( $C_{2}$ ).

*Hypothesis 3*: Due to the institutional environment of economic decentralization and political centralization in China, together with the fact that the major evaluation indicators and the promotion of local government officials depend on regional economic progress, there may generate a rent-seeking motive even collusion between local governments and enterprises (Nie and Li, 2013; Jia and Nie, 2017; Utama et al., 2019). Local governments are the direct supervising agencies and they pay the price for work-safety supervision literally. Local governments have two strategies. They may implement work-safe policies strictly and supervise enterprises strictly with high administrative expenditure or they may supervise enterprises perfunctorily with a relatively low administrative expenditure based on the assumption that accidents and injuries happen with probability by chance. Notably, the central government represents the interests of the whole public and it’s the major beneficiary of work-safe policy implementation. From this point of view, the central government has only one strategy (i.e., inspecting local governments strictly to promote local governments’ supervision). Given this, we consider the influences of the central government through *R*_1_, *R*_2_. Specifically, assuming a local government adopts the strict supervision strategy, the overall work-safety level of enterprises in the area has been improved. Then, material rewards (such as economic support) and immaterial rewards (such as promotion of local officials) are possible gained from the central government. When local governments follow strict supervision and enterprises choose to purchase work safety service, the reward income given by the central government to local governments is quantified as $R_{1}$ . When local governments follow strict supervision and enterprises choose not to buy work safety service, the reward income given by the central government is quantified as $R_{2}$ . It is obvious that $R_{1}>R_{2}$ .

*Hypothesis 4*: Benefits gained from work-safety-service purchase include two parts: impairment income (i.e., with professional work safety expertise and technology of service organizations, the losses can be reduced by internal risks identification and elimination in enterprises before accidents and injuries) and gain income (i.e., output value increased by improving working condition and productivity). No matter impairment income or gain income, they are difficult to turn into materialized products and asses the actual value. Noted that value of work safety service is usually hard to manifest immediately, enterprises are not proactive enough to purchase work safety service (Sinclair et al., 2013). It is assumed in this paper that the strategy space of enterprises is {purchase, non-purchase}, denoted as { $B_{1},B_{2}$ }. When enterprises choose to purchase work safety service, they may get some preferential policies from local governments, such as economic subsidies, tax incentive and financial support, etc., quantified as A and work-safety-service cost is $C_{0}$ . We assume the accident rate under this circumstance is $\theta_{1}$ and the punishment from the local government is quantified as loss F , and the loss of the enterprise itself is L . It’s important to note that when accidents happen in enterprises, injuries occur and workers will ask for compensation; if the damage is awfully bad, maybe leading enterprises to a suspension, this will be a subsequent loss both in time and economy. We consider compensation and time-economy loss inside the enterprises as L . Meanwhile, after the accidents and injuries happening in enterprises, the local government goes to the enterprise for accident investigation and violation punishments, which leads to a loss F . Notably, L is different from F . L refers to a loss inside the enterprises and F is a loss penalized by local governments outside the enterprises, and F is regarded as part of administrative revenue to local governments. When the enterprise chooses not to purchase work safety service, the local government may discover the violations if it performs strict supervision. Then, the enterprise will face punishments such as suspension and rectification, quantified as loss u . Assuming the accident rate of the enterprise under this circumstance is $\theta_{2}$ , we have $\theta_{2}>\theta_{1}>0$ .

*Hypothesis 5*: When the local government chooses loose supervision and accidents really happen in the enterprise, the central government, out of the need of transferring political and social pressure, will make the local government accountable for poor supervision and punish them (Sun et al., 2021). Relevant officials of the local government are probably prosecuted or even suspended for investigation; the punishment will be quantified as the local government loss G . The punishments borne by the local government due to loose supervision is also related to the strategy and accident rate of enterprises. When the enterprise chooses to purchase work safety service, the punishment borne by the local government is quantified as $\theta_{1}G$ . When the enterprise chooses not to purchase work safety service, the punishment that the local government should bear is quantified as $\theta_{2}G$ . It is evident that $\theta_{2}G>\theta_{1}G>0$ .

### Model building

Strategy evolution between the local government and the enterprise turns out to be a constant adjusting biological dynamic process, since both participants in the game have limited rationality when they choose their own strategy. According to the above assumptions, the game payoffs matrix between the local government and the enterprise is constructed, as shown in Table 1. We repeatedly choose one participant from local governments and one participant from enterprises, and then we make them contest to discover their payoffs in a single game. Each participant is allowed to choose a strategy once in the single game and make strategy adjustment in next single game. Suppose in the initial stage of the game, the proportion of local governments opting for strict supervision is x , and the initial proportion of enterprises opting for work-safety-service purchase is y .

**Table 1:** Game payoffs matrix between the local government and the enterprise.

Local government	Enterprise	
	$\left(B_{1}\right)$ Purchase of service (y)	$\left(B_{2}\right)$ Non purchase of service (1−y)
$\left(A_{1}\right)$ Strict supervision (x)	$R_{1}-C_{1}-A-C_{2}+\theta_{1}F$ , $A-C_{0}-\theta_{1}\left(F+L\right)$	$R_{2}-C_{1}-C_{2}+\theta_{2}F+u$ , $-\theta_{2}\left(F+L\right)-u$
$\left(A_{2}\right)$ Loose supervision (1−x)	$-C_{2}-\theta_{1}G+\theta_{1}F$ , $-C_{0}-\theta_{1}\left(F+L\right)$	$-C_{2}-\theta_{2}G+\theta_{2}F$ , $-\theta_{2}\left(F+L\right)$

## Evolutionary equilibrium analysis

We calculate the possible equilibrium points in the evolution process according to the game payoffs matrix in Table 1, and then discuss the stability of each equilibrium point according to the possible range of each parameter.

### Equilibrium points in the evolution process

According to the assumptions above, any point (x,y) within the region [0,1]×[1,0] can be used to represent the status S={(x,1−x),(y,1−y)} (i.e., the evolutionary dynamics of local government’s strategy and enterprise’s strategy). Based on the method of replicator dynamics analysis and evolutionary game (Friedman, 1991), the fitness of different strategies of local governments and enterprises is solved.

(1) Let the expected benefit of local governments when adopting strict supervision $\left(A_{1}\right)$ and loose supervision $\left(A_{2}\right)$ be $f_{1}^{A_{1}}$ and $f_{1}^{A_{2}}$ , and the average fitness is $\bar{f_{1}}$ .

(1) $$\begin{array}{c} f_{1}^{A_{1}}=y\left(R_{1}-C_{1}-A-C_{2}+\theta_{1}F\right)+\\ \left(1-y\right)\left(R_{2}-C_{1}-C_{2}+\theta_{2}F+u\right) \end{array}$$

(2) $$f_{1}^{A_{2}}=y\left(-C_{2}-\theta_{1}G+\theta_{1}F\right)+\left(1-y\right)\left(-\theta_{2}G+\theta_{2}F-C_{2}\right)$$

(3) $$\bar{f_{1}}=xf_{1}^{A_{1}}+\left(1-x\right)f_{1}^{A_{2}}$$

(4) $$\begin{array}{l} F\left(x\right)=x\left(f_{1}^{A_{1}}-\bar{f_{1}}\right)=\\ x\left(1-x\right)\left\{\begin{array}{l} \left[\left(\theta_{1}-\theta_{2}\right)G+R_{1}-R_{2}-A-u\right]y+\\ \theta_{2}G+R_{2}-C_{1}+u \end{array}\right\} \end{array}$$

(5) $$\begin{array}{l} LetF\left(x\right)=0,\mathrm{and}\;\mathrm{we}\;\mathrm{find}x=0,x=1,\\ y^{*}=\frac{\theta_{2}G+R_{2}-C_{1}+u}{\left(\theta_{2}-\theta_{1}\right)G+R_{2}-R_{1}+A+u} \end{array}$$

(2) Let the expected benefit of enterprises when adopting service purchase $\left(B_{1}\right)$ and service non-purchase $\left(B_{2}\right)$ be $f_{2}^{B_{1}}$ and $f_{2}^{B_{2}}$ , and the average fitness is $\bar{f_{2}}$ .

(6) $$\begin{array}{c} f_{2}^{B_{1}}=x\left[A-C_{0}-\theta_{1}\left(F+L\right)\right]+\\ \left(1-x\right)\left[-C_{0}-\theta_{1}\left(F+L\right)\right] \end{array}$$

(7) $$f_{2}^{B_{2}}=x\left[-\theta_{2}\left(F+L\right)-u\right]+\left(1-x\right)\left[-\theta_{2}\left(F+L\right)\right]$$

(8) $$\bar{f_{2}}=yf_{2}^{B_{1}}+\left(1-y\right)f_{2}^{B_{2}}$$

(9) $$\begin{array}{l} F\left(y\right)=y\left(f_{2}^{B_{1}}-\bar{f_{2}}\right)=\\ y\left(1-y\right)\left[\left(A+u\right)x-\left(\theta_{1}-\theta_{2}\right)\left(F+L\right)-C_{0}\right] \end{array}$$

(10) $$\begin{array}{l} \mathrm{Let}F\left(y\right)=0,\mathrm{and}\;\mathrm{we}\;\mathrm{find}y=0,\\ y=1,x^{*}=\frac{-\left(\theta_{2}-\theta_{1}\right)\left(F+L\right)+C_{0}}{A+u} \end{array}$$

According to Equations 5 and 10, five equilibrium points of this dynamic system are obtained, which are O(0,0) , U(1,0) , V(1,1) , W(0,1) and $E\left(x^{*},y^{*}\right)$ . The first four points are unconditional equilibrium points. If and only if $E\left(x^{*},y^{*}\right)\in \left[0,1\right]\times \left[1,0\right]$ , $E\left(x^{*},y^{*}\right)$ is also the equilibrium point of the system.

### Stability analysis of equilibrium points

Replicator dynamics is actually a dynamic differential equation that describes the frequency of a particular strategy being adopted in a population. Based on the principles of evolution, a strategy whose benefits or payoffs are higher than the average fitness of the population will develop in the population (i.e., survival of the fittest), as reflected in the growth rate of the strategy (replicator dynamic equation) greater than zero (Friedman, 1991). The stability of the equilibrium points can be derived by analyzing the Jacobian matrix based on the method mentioned in literature (Friedman, 1991). The Jacobian matrix of this system is denoted as J , whose Jacobian determinant is recorded as det(J) (abbreviated as D ) and whose trace is recorded as Tr(J) (abbreviated as T ). According to Equations 5 and 10, the Jacobian matrix of the system can be obtained as J .


(11) $$\begin{array}{l} J=\left[\begin{array}{cc} \frac{\partial F\left(x\right)}{\partial x} & \frac{\partial F\left(x\right)}{\partial y}\\ \frac{\partial F\left(y\right)}{\partial x} & \frac{\partial F\left(y\right)}{\partial y} \end{array}\right]\\ =\left[\begin{array}{cc} \left(1-2x\right)\left\{\begin{array}{l} \left[\begin{array}{l} \left(\theta_{1}-\theta_{2}\right)G+\\ R_{1}-R_{2}-\\ A-u \end{array}\right]y+\\ \theta_{2}G+R_{2}-C_{1}+u \end{array}\right\} & x\left(1-x\right)\left[\begin{array}{l} \left(\theta_{1}-\theta_{2}\right)G+\\ R_{1}-\\ R_{2}-A-u \end{array}\right]\\ y\left(1-y\right)\left(A+u\right) & \left(1-2y\right)\left[\begin{array}{l} \left(A+u\right)x-\\ \left(\theta_{1}-\theta_{2}\right)\\ \left(F+L\right)-C_{0} \end{array}\right] \end{array}\right] \end{array}$$

(12) $$\begin{array}{l} D=Det\left(J\right)=\left(1-2x\right)\left(1-2y\right)\left[\left(A+u\right)x-\left(\theta_{1}-\theta_{2}\right)\left(F+L\right)-C_{0}\right]\\ \times \left\{\left[\left(\theta_{1}-\theta_{2}\right)G+R_{1}-R_{2}-A-u\right]y+\theta_{2}G+R_{2}-C_{1}+u\right\}\\ -xy\left(1-x\right)\left(1-y\right)\left(A+u\right)\left[\left(\theta_{1}-\theta_{2}\right)G+R_{1}-R_{2}-A-u\right] \end{array}$$

(13) $$\begin{array}{l} T=Tr\left(J\right)=\left(1-2x\right)\left\{\begin{array}{l} \left[\left(\theta_{1}-\theta_{2}\right)G+R_{1}-R_{2}-A-u\right]y+\\ \theta_{2}G+R_{2}-C_{1}+u \end{array}\right\}\\ -\left(1-2y\right)\left[\left(A+u\right)x-\left(\theta_{1}-\theta_{2}\right)\left(F+L\right)-C_{0}\right] \end{array}$$

The stability of the equilibrium points is determined by the determinant and trace values of the Jacobian matrix. It’s adequate and necessary for an equilibrium point to be an evolutionary stable point if det(J)>0,Tr(J)<0 , (i.e., D>0,T<0 ). According to the assumptions and parameter settings in the model, the stability of each equilibrium point is discussed in the following three cases (12 specific situations in total).

**Figure 1:**
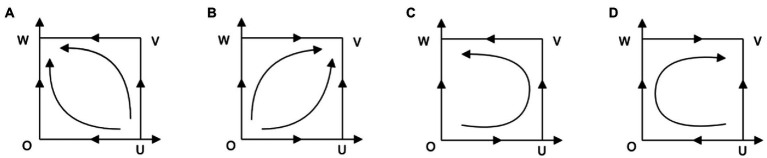
Phase diagram of system evolution when $\left(\theta_{2}-\theta_{1}\right)\left(F+L\right)+A+u-C_{0}>\left(\theta_{2}-\theta_{1}\right)\left(F+L\right)-C_{0}>0$ .

(1) Scenario 1: $\left(\theta_{2}-\theta_{1}\right)\left(F+L\right)+A+u-C_{0}>\left(\theta_{2}-\theta_{1}\right)\left(F+L\right)-C_{0}>0$ . The local stability of each equilibrium point is shown in Table 2 (EPs is short for equilibrium points). According to the analysis in Table 2, the corresponding system evolution phase diagram is drawn, as shown in Figure 1.

**Table 2:** Stability analysis of equilibrium points when $\left(\theta_{2}-\theta_{1}\right)\left(F+L\right)+A+u-C_{0}>\left(\theta_{2}-\theta_{1}\right)\left(F+L\right)-C_{0}>0$ .

Situations	EPs					
	Parameter range	O(0,0)	W(0,1)	U(1,0)	V(1,1)	$E\left(x^{*},y^{*}\right)$
A	$\begin{array}{l} \theta_{2}G+R_{2}+u-C_{1}<0;\\ \theta_{1}G+R_{1}-A-C_{1}<0;\\ \left(\theta_{2}-\theta_{1}\right)\left(F+L\right)-C_{0}\\ +\theta_{1}G+R_{1}-A-C_{1}<0;\\ \theta_{2}G+R_{2}+u-C_{1}\\ +\left(\theta_{2}-\theta_{1}\right)\left(F+L\right)\\ +A+u-C_{0}<0. \end{array}$	D < 0 Saddle point	D > 0, T < 0 ESS	D > 0, T > 0 Unstable point	D < 0 Saddle point	——
B	$\begin{array}{l} \theta_{2}G+R_{2}+u-C_{1}>0;\\ \theta_{1}G+R_{1}-A-C_{1}>0;\\ \theta_{2}G+R_{2}+u-C_{1}\\ -\left(\theta_{2}-\theta_{1}\right)\left(F+L\right)\\ +C_{0}>0;\\ -\left(\theta_{1}G+R_{1}-A-C_{1}\right)\\ +\left(\theta_{2}-\theta_{1}\right)\left(F+L\right)\\ +A+u-C_{0}<0. \end{array}$	D > 0, T > 0 Unstable point	D < 0 Saddle point	D < 0 Saddle point	D > 0, T < 0 ESS	——
C	$\begin{array}{l} \theta_{2}G+R_{2}+u-C_{1}>0;\\ \theta_{1}G+R_{1}-A-C_{1}<0;\\ \theta_{2}G+R_{2}+u-C_{1}\\ -\left(\theta_{2}-\theta_{1}\right)\left(F+L\right)\\ +C_{0}>0;\\ \left(\theta_{2}-\theta_{1}\right)\left(F+L\right)-C_{0}\\ +\theta_{1}G+R_{1}-A-C_{1}<0. \end{array}$	D > 0, T > 0 Unstable point	D > 0, T < 0 ESS	D < 0 Saddle point	D < 0 Saddle point	——
D	$\begin{array}{l} \theta_{2}G+R_{2}+u-C_{1}<0;\\ \theta_{1}G+R_{1}-A-C_{1}>0;\\ \theta_{2}G+R_{2}+u-C_{1}\\ +\left(\theta_{2}-\theta_{1}\right)\left(F+L\right)\\ +A+u-C_{0}<0;\\ -\left(\theta_{1}G+R_{1}-A-C_{1}\right)\\ +\left(\theta_{2}-\theta_{1}\right)\left(F+L\right)\\ +A+u-C_{0}<0. \end{array}$	D < 0 Saddle point	D < 0 Saddle point	D > 0, T > 0 Unstable point	D > 0, T < 0 ESS	——

System phase analysis: When $\left(\theta_{2}-\theta_{1}\right)\left(F+L\right)+A+u-C_{0}>\left(\theta_{2}-\theta_{1}\right)\left(F+L\right)-C_{0}>0$ , the payoff with work-safety-service purchase for enterprises is dominantly higher than the payoff with work-safety-service non-purchase no matter what strategy local governments choose. Therefore, for enterprises, their evolutionary stable strategy is “purchase service,” that is, y→1 . It implies desired scenarios in reality in which work-safety-service organizations are probably providing work safety service with high standard and with which enterprises can reduce risks greatly and improve work safety condition substantially. As is shown in Figure 1, the evolutionary stable points are the same (i.e., W(0,1) ) in Figures 1A,C, since under these two circumstances, local governments get higher payoff with strategy “loose supervision” when enterprises choose “purchase service.” Therefore, x→0 . On the contrary, when $\theta_{1}G+R_{1}-A-C_{1}>0$ , the payoff with strict supervision for local governments is dominantly higher, that is, x→1 as is shown in Figures 1B,D. It should be noted that in this case, point $E\left(x^{*},y^{*}\right)\notin \left[0,1\right]\times \left[1,0\right]$ , so it is not discussed here.

**Figure 2:**
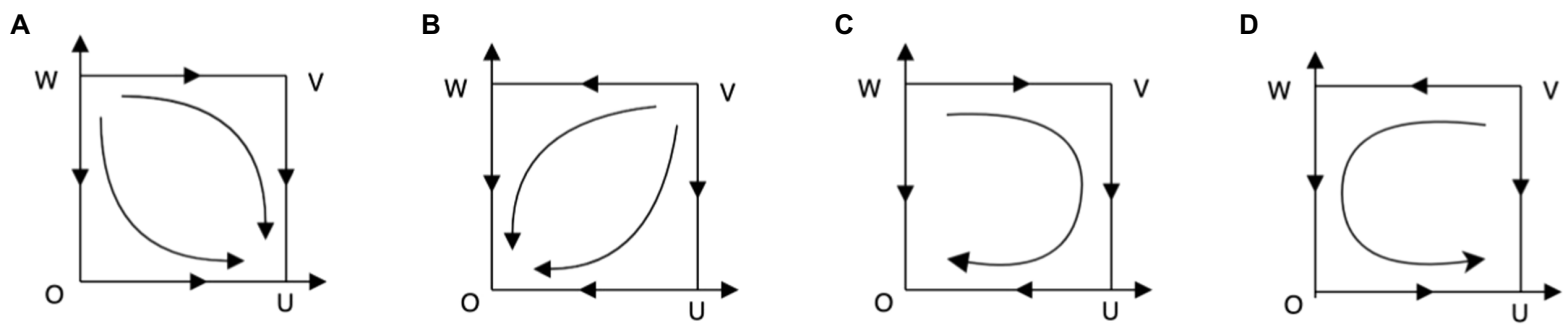
Phase diagram of system evolution when $\left(\theta_{2}-\theta_{1}\right)\left(F+L\right)-C_{0}<\left(\theta_{2}-\theta_{1}\right)\left(F+L\right)+A+u-C_{0}<0$ .

(2) Scenario 2: $\left(\theta_{2}-\theta_{1}\right)\left(F+L\right)-C_{0}<\left(\theta_{2}-\theta_{1}\right)\left(F+L\right)+A+u-C_{0}<0$ . The local stability of each equilibrium point is shown in Table 3 (EPs is short for equilibrium points). According to the analysis in Table 3, the corresponding system evolution phase diagram is drawn, as shown in Figure 2.

**Table 3:** Stability analysis of equilibrium points for $\left(\theta_{2}-\theta_{1}\right)\left(F+L\right)-C_{0}<\left(\theta_{2}-\theta_{1}\right)\left(F+L\right)+A+u-C_{0}<0$ .

Situations	Eps					
	Parameter range	O(0,0)	W(0,1)	U(1,0)	V(1,1)	$E\left(x^{*},y^{*}\right)$
A	$\begin{array}{l} \theta_{2}G+R_{2}+u-C_{1}>0;\\ \theta_{1}G+R_{1}-A-C_{1}>0;\\ \left(\theta_{2}-\theta_{1}\right)\left(F+L\right)-C_{0}\\ +\theta_{1}G+R_{1}-A-C_{1}>0;\\ \theta_{2}G+R_{2}+u-C_{1}\\ +\left(\theta_{2}-\theta_{1}\right)\left(F+L\right)\\ +A+u-C_{0}>0. \end{array}$	D < 0 Saddle point	D > 0, T > 0 Unstable point	D > 0, T < 0 ESS	D < 0 saddle point	——
B	$\begin{array}{l} \theta_{2}G+R_{2}+u-C_{1}<0;\\ \theta_{1}G+R_{1}-A-C_{1}<0;\\ \theta_{2}G+R_{2}+u-C_{1}\\ -\left(\theta_{2}-\theta_{1}\right)\left(F+L\right)\\ +C_{0}<0;\\ -\left(\theta_{1}G+R_{1}-A-C_{1}\right)\\ +\left(\theta_{2}-\theta_{1}\right)\left(F+L\right)\\ +A+u-C_{0}>0. \end{array}$	D > 0, T < 0 ESS	D < 0 saddle point	D < 0 Saddle point	D > 0, T > 0 Unstable point	——
C	$\begin{array}{l} \theta_{2}G+R_{2}+u-C_{1}<0;\\ \theta_{1}G+R_{1}-A-C_{1}>0;\\ \theta_{2}G+R_{2}+u-C_{1}\\ -\left(\theta_{2}-\theta_{1}\right)\left(F+L\right)\\ +C_{0}<0;\\ \left(\theta_{2}-\theta_{1}\right)\left(F+L\right)-C_{0}\\ +\theta_{1}G+R_{1}-A-C_{1}>0. \end{array}$	D > 0, T < 0 ESS	D > 0, T > 0 Unstable point	D < 0 Saddle point	D < 0 saddle point	——
D	$\begin{array}{l} \theta_{2}G+R_{2}+u-C_{1}>0;\\ \theta_{1}G+R_{1}-A-C_{1}<0;\\ \theta_{2}G+R_{2}+u-C_{1}\\ +\left(\theta_{2}-\theta_{1}\right)\left(F+L\right)\\ +A+u-C_{0}>0;\\ -\left(\theta_{1}G+R_{1}-A-C_{1}\right)\\ +\left(\theta_{2}-\theta_{1}\right)\left(F+L\right)\\ +A+u-C_{0}>0. \end{array}$	D < 0 Saddle point	D < 0 Saddle point	D > 0, T < 0 ESS	D > 0, T > 0 Unstable point	——

System phase analysis: When $\left(\theta_{2}-\theta_{1}\right)\left(F+L\right)-C_{0}<\left(\theta_{2}-\theta_{1}\right)\left(F+L\right)+A+u-C_{0}<0$ , it means the payoff with work-safety-service purchase for enterprises is dominantly lower than the payoff with work-safety-service non-purchase no matter what strategy local governments choose. Therefore, for enterprises, their evolutionary stable strategy is “nonpurchase service,” that is y→0 . It implies undesired scenarios in reality in which work-safety-service organizations are not well developed and work-safety-service market hardly exists since work-safety-service demand is not sufficient to support a profession. As is shown in Figure 2, the evolutionary stable points are the same (i.e., U(1,0) ) in Figures 1A,D, since under these two circumstances, local governments get higher payoff with strategy “strict supervision” when enterprises choose “nonpurchase service.” Therefore, x→1 . On the contrary, when $\theta_{2}G+R_{2}+u-C_{1}<0$ , the payoff with loose supervision for local governments is dominantly higher, that is, x→0 as is shown in Figures 1B,C. It should be noted that in this case, point $E\left(x^{*},y^{*}\right)\notin \left[0,1\right]\times \left[1,0\right]$ , so it is not discussed here.

**Figure 3:**
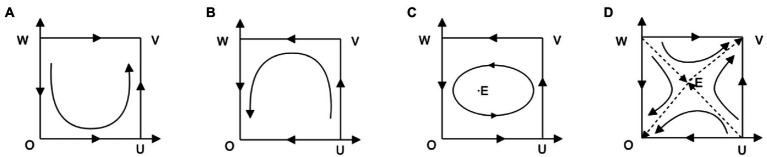
Phase diagram of system evolution for $\left(\theta_{2}-\theta_{1}\right)\left(F+L\right)-C_{0}<0<\left(\theta_{2}-\theta_{1}\right)\left(F+L\right)+A+u-C_{0}$ .

(3) Scenario 3: $\left(\theta_{2}-\theta_{1}\right)\left(F+L\right)-C_{0}<0<\left(\theta_{2}-\theta_{1}\right)\left(F+L\right)+A+u-C_{0}$ . The local stability of each equilibrium point is shown in Table 4 (EPs is short for equilibrium points). According to the analysis in Table 4, the corresponding system evolution phase diagram is drawn, as shown in Figure 3.

**Table 4:** Stability analysis of equilibrium points when $\left(\theta_{2}-\theta_{1}\right)\left(F+L\right)-C_{0}<0<\left(\theta_{2}-\theta_{1}\right)\left(F+L\right)+A+u-C_{0}$ .

Situations	EPs					
	Parameter range	O(0,0)	W(0,1)	U(1,0)	V(1,1)	$E\left(x^{*},y^{*}\right)$
A	$\begin{array}{l} \theta_{2}G+R_{2}+u-C_{1}>0;\\ \theta_{1}G+R_{1}-A-C_{1}>0;\\ \left(\theta_{2}-\theta_{1}\right)\left(F+L\right)-C_{0}\\ +\theta_{1}G+R_{1}-A-C_{1}>0;\\ -\left(\theta_{2}G+R_{2}+u-C_{1}\right)\\ +\left(\theta_{2}-\theta_{1}\right)\left(F+L\right)\\ +A+u-C_{0}<0. \end{array}$	D < 0 Saddle point	D > 0, T > 0 Unstable point	D < 0 Saddle point	D > 0, T < 0 ESS	——
B	$\begin{array}{l} \theta_{2}G+R_{2}+u-C_{1}<0;\\ \theta_{1}G+R_{1}-A-C_{1}<0;\\ \theta_{2}G+R_{2}+u-C_{1}\\ -\left(\theta_{2}-\theta_{1}\right)\left(F+L\right)\\ +C_{0}<0;\\ \theta_{2}G+R_{2}+u-C_{1}\\ +\left(\theta_{2}-\theta_{1}\right)\left(F+L\right)\\ +A+u-C_{0}<0. \end{array}$	D > 0, T < 0 ESS	D < 0 Saddle point	D > 0, T > 0 Unstable point	D < 0 Saddle point	——
C	$\begin{array}{l} \theta_{2}G+R_{2}+u-C_{1}>0;\\ \theta_{1}G+R_{1}-A-C_{1}<0. \end{array}$	D < 0 Saddle point	D < 0 Saddle point	D < 0 Saddle point	D < 0 Saddle point	D > 0, T = 0 Unstable point
D	$\begin{array}{l} \theta_{2}G+R_{2}+u-C_{1}<0;\\ \theta_{1}G+R_{1}-A-C_{1}>0. \end{array}$	D > 0, T < 0 ESS	D > 0, T > 0 Unstable point	D > 0, T > 0 Unstable point	D > 0, T < 0 ESS	D < 0, T = 0 Saddle point

System phase analysis: According to the parameters range in Table 4a, when $\theta_{2}G+R_{2}+u-C_{1}>0$ and $\theta_{1}G+R_{1}-A-C_{1}>0$ , the payoff with strict supervision for local governments is dominantly higher than the payoff with loose supervision no matter what strategy enterprises choose. Therefore, we always have x→1 . When the local government chooses “strict supervision,” the payoff of enterprises with “purchase service” is dominantly higher than that with “nonpurchase service,” so y→1 . Evolutionary trend is shown in Figure 3A. According to the parameters range in Table 4b, when $\theta_{2}G+R_{2}+u-C_{1}<0$ and $\theta_{1}G+R_{1}-A-C_{1}<0$ , the payoff with loose supervision for local governments is dominantly higher than the payoff with strict supervision no matter what strategy enterprises choose. Therefore, we always have x→0 . When local governments choose “loose supervision,” enterprises can only get higher payoff if they choose “nonpurchase service,” so y→0 . The evolutionary trend is shown in Figure 3B. According to the parameters range in Table 4c, when $\left(\theta_{2}-\theta_{1}\right)\left(F+L\right)+A+u-C_{0}>0$ and local governments choose “strict supervision,” the best strategy for enterprises is “purchase service”; when $\left(\theta_{2}-\theta_{1}\right)\left(F+L\right)-C_{0}<0$ and local governments choose “loose supervision,” the best strategy for enterprises is “nonpurchase service”; when $\theta_{1}G+R_{1}-A-C_{1}<0$ and enterprises choose to “purchase service,” the best strategy for the local governments is “loose supervision”; when $\theta_{2}G+R_{2}+u-C_{1}>0$ and enterprises choose not to purchase service, the best strategy for local governments is “strict supervision.” It is tricky to find out in Figure 3C that the strategies of both participants are dynamically and constantly changing, and the system cannot achieve a stable point. When $\left(\theta_{2}-\theta_{1}\right)\left(F+L\right)-C_{0}<0<\left(\theta_{2}-\theta_{1}\right)\left(F+L\right)+A+u-C_{0}$ , $\left(\theta_{2}-\theta_{1}\right)\left(F+L\right)+A+u-C_{0}>0$ and $\theta_{1}G+R_{1}-A-C_{1}>0$ , there exists two evolutionary trends. One is the desired trend in which the best strategy for local governments is strict supervision and the best response for enterprises is work-safety-service purchase, and vice versa. The other is the undesired trend in which the best strategy for local governments is loose supervision and the best response for enterprises is work-safety-service non-purchase, and vice versa. These evolutionary trends give us a clue to think hard on how to make local governments supervise strictly and enterprises purchase work-safety-service.

### Influence of parameter variations on evolution results

Based on the stability analysis of equilibrium points above, it can be seen that in Figure 3D, the system has two evolutionary stable points, which are O(0,0) and V(1,1) . Under this circumstance, the variations of different parameters in the payoff matrix of both participants may lead the system to different evolutionary stable points, but to which evolutionary stable point needs further discussion.

In Figure 3D, the coordinates of saddle point $E\left(x^{*},y^{*}\right)$ are $x^{*}=\frac{-\left(\theta_{2}-\theta_{1}\right)\left(F+L\right)+C_{0}}{A+u}$ and $y^{*}=\frac{\theta_{2}G+R_{2}-C_{1}+u}{\left(\theta_{2}-\theta_{1}\right)G+R_{2}-R_{1}+A+u}$ . Let the area of quadrilateral EUVW be $S^{1}$ , and the area of quadrilateral EWOU be $S^{2}$ . When $S^{1}>S_{2}$ , the system is more likely to converge to V(1,1) than O(0,0) . This is the desired evolutionary path. On the contrary, the system is more likely to converge to O(0,0) , which is an undesired situation.


(14) $$\begin{array}{l} S^{2}=\frac{1}{2}\left(x^{*}+y^{*}\right)=\\ \frac{1}{2}\left(\begin{array}{l} \frac{-\left(\theta_{2}-\theta_{1}\right)\left(F+L\right)+C_{0}}{A+u}+\\ \frac{\theta_{2}G+R_{2}-C_{1}+u}{\left(\theta_{2}-\theta_{1}\right)G+R_{2}-R_{1}+A+u} \end{array}\right);S^{1}=1-\frac{1}{2}\left(x^{*}+y^{*}\right) \end{array}$$

There are 12 parameters involved in the model. This paper mainly considers the effect of local government’s ex ante and ex post supervision, and effect of central government’s inspection on local governments, so the parameters $G,R_{1},R_{2},F,A,u,C_{1}$ are selected to investigate the influence on the system evolution results. According to the above analysis and Equation 14, the influence of the selected parameter on the convergence result is estimated. The influence of parameter change on evolution results is shown in Table 5.

**Table 5:** Influence of parameter change on evolution results.

Parameters	G↑	$C_{1}↑$	$R_{1}↑$	$R_{2}↑$	A↑	F↑	u↑
$S^{1}$	↑	‑	↑	↑	Unsteady	↑	↑
Stable point	V(1,1)	O(0,0)	V(1,1)	V(1,1)	Unsteady	V(1,1)	V(1,1)

## Numerical experiments and simulation results analysis

In this section, Matlab 2014b is utilized to explore the impact of a change in the value of key parameters on the probability of different strategy choices and evolutionary paths among participants. According to the assumption, relevant parameters should meet the following criteria: $\left(\theta_{2}-\theta_{1}\right)\left(F+L\right)-C_{0}<0<\left(\theta_{2}-\theta_{1}\right)\left(F+L\right)+A+u-C_{0}$ ; $\theta_{2}G+R_{2}+u-C_{1}\langle 0;\theta_{1}G+R_{1}-A-C_{1}\rangle 0$ .

(1) Influence of the initial proportion variations of a certain strategy on the evolution result.

**Figure 4:**
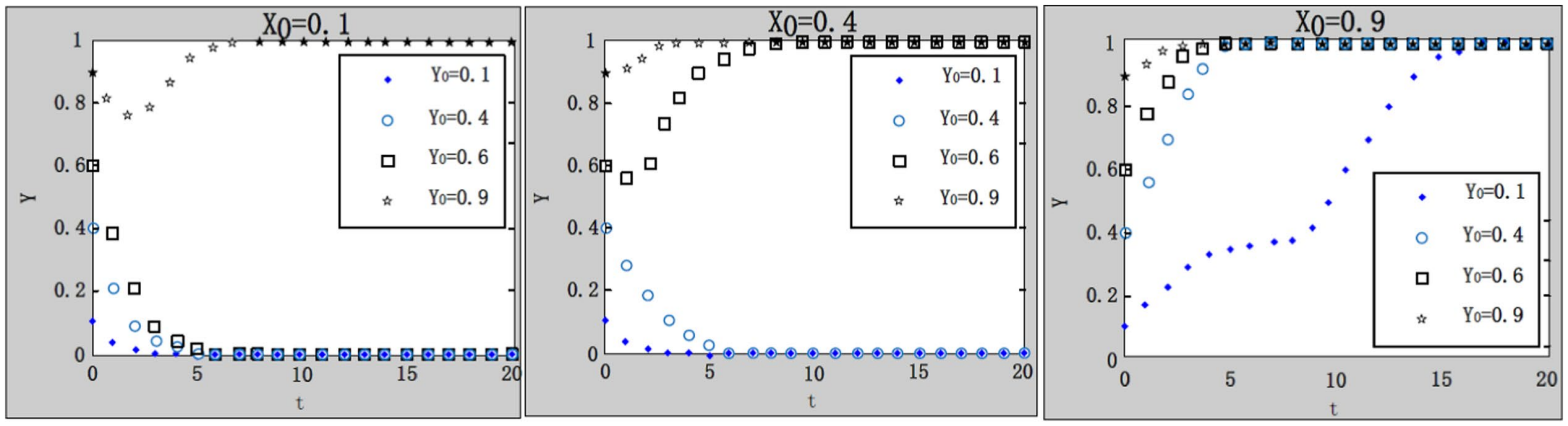
Influence of different values of initial proportion on system evolution.

We take $G=1.1;R_{1}=4.9;R_{2}=1;F=4;A=1;u=1;C_{1}=4;L=5;C_{0}=8.3;\theta_{1}=0.1;\theta_{2}=0.9$ , and consider the different initial proportions ( $x_{0}=0.1;0.4;0.9$ ) of “strict supervision” by the local government. The evolutionary trend is shown in Figure 4. The results indicate that with the increase of the initial proportion of “strict supervision” of local governments, more and more enterprises choose the strategy “purchase service.” It becomes faster when system converges to evolutionary stable point.

(2) Influence of economic subsidies (or preferential policies) given by local governments on the evolution result.

**Figure 5:**
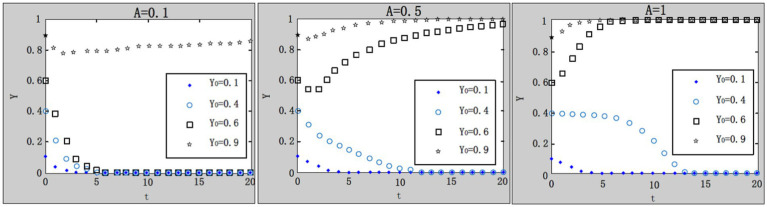
Influence of local government subsidies on system evolution.

We take $\begin{array}{l} x_{0}=0.4;G=1.1;R_{1}=4.9;R_{2}=1;F=4;u=1;\\ C_{1}=4;L=5;C_{0}=8.3;\theta_{1}=0.1;\theta_{2}=0.9 \end{array}$ . According to the parameter settings, the values of A=0.1;0.5;1 are selected, respectively, and the results are shown in Figure 5.

Within the range of subsidies and support from local governments, the increase of subsidies has little effect on the evolution trend of the system. A possible explanation is that although local governments try best to subsidize enterprises and encourage enterprises to purchase work safety service, the subsidy or support is considerably inadequate compared with the investment of enterprises (in this case $A_{\max}=1;C_{0}=8.3$ ). It is concluded that local government’s subsidies and support is not sufficient to motivate enterprises to purchase work safety service. For example, according to the Implementation Plan of Special Subsidy Fund for the Reconstruction of Automatic Control System of Major Dangerous Chemicals issued by Ruian City, Zhejiang Province, enterprises can get a subsidy of 50,000 yuan with work safety investment between 200,000 and 300,000 yuan; and they can get a subsidy of 80,000yuan with work safety investment above 300,000 yuan (Kong et al., 2015). It is inferred that the current subsidy ratio and amount are not high, which has little incentive effect on enterprises.

(3) Influence of local government’s ex post penalties on the evolution result.

**Figure 6:**
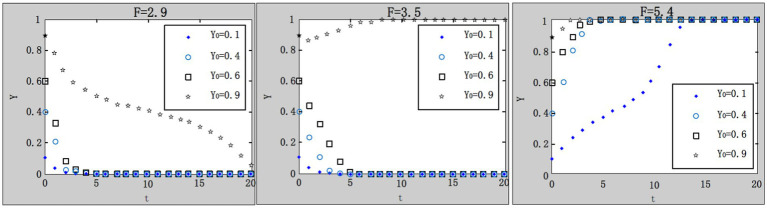
Influence of local government’s ex post penalties on system evolution.

We take $\begin{array}{l} x_{0}=0.4;G=1.1;R_{1}=4.9;R_{2}=1;A=1;u=1;\\ C_{1}=4;L=5;C_{0}=8.3;\theta_{1}=0.1;\theta_{2}=0.9 \end{array}$ . According to the parameter settings, the values of F=2.9;3.5;5.4 are selected, respectively, and the results are shown in Figure 6. Within the range of government penalties, as the penalties on enterprises increases, the system gradually evolves to the desired evolutionary stable point.

(4) Influence of local government’s ex ante penalties on the evolution result.

**Figure 7:**
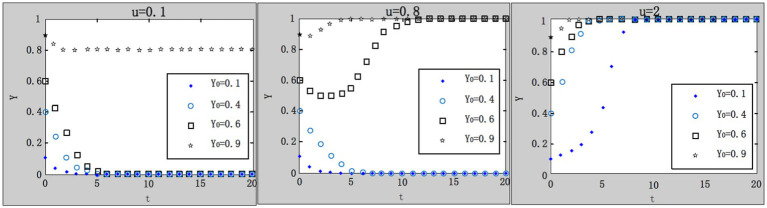
Influence of local government’s ex ante penalties on system evolution.

We take $\begin{array}{l} x_{0}=0.4;G=1.1;R_{1}=4.9;R_{2}=1;A=1;\\ F=4;C_{1}=4;L=5;C_{0}=8.3;\theta_{1}=0.1;\theta_{2}=0.9 \end{array}$ . According to the parameter settings, the values of u=0.1;0.8;2 are selected, respectively, and the results are shown in Figure 7.

Within the range of government penalties, as the ex ante penalties on enterprises increases, the system gradually evolves to the desired evolutionary stable point. The convergence speed of the system under ex ante penalties is faster than that under ex post penalties, which indicates ex ante supervision has a greater stimulation on enterprises in increasing work safety investment and work-safety-service purchase.

(5) Influence of local government’s ex ante supervision cost on the evolution result.

**Figure 8:**
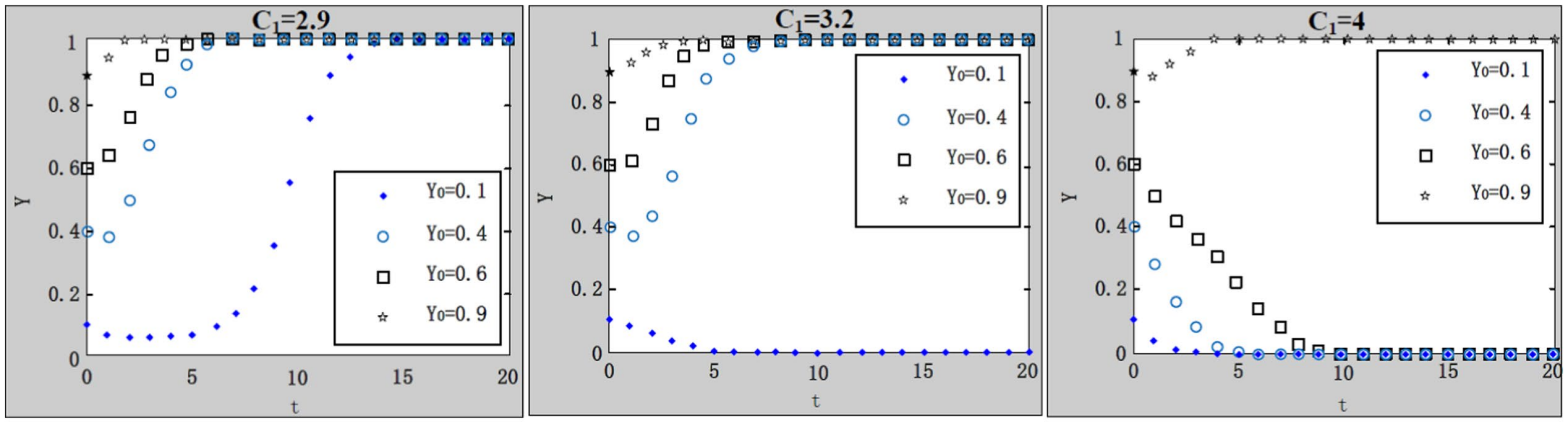
Influence of local government’s ex ante supervision cost on system evolution.

We take $\begin{array}{l} x_{0}=0.4;G=1.1;R_{1}=4.9;R_{2}=1;A=1;\\ F=4;u=1;L=5;C_{0}=8.3;\theta_{1}=0.1;\theta_{2}=0.9 \end{array}$ . According to the parameter settings, the values of $C_{1}=2.9;3.2;4$ are selected, respectively, and the results are shown in Figure 8. Within the range of local government’s ex ante supervision cost, as the cost increases, the system gradually evolves into an undesired evolutionary stable point. It indicates that local governments may slack off on ex ante supervision with less daily inspection and random inspection on enterprises due to the increasing ex ante supervision cost. Therefore, it is necessary for the central government to urge local governments to improve their motivation and initiative of ex ante supervision.

(6) Influence of the central government’s penalties on local government’s loose supervision on the evolution result.

**Figure 9:**
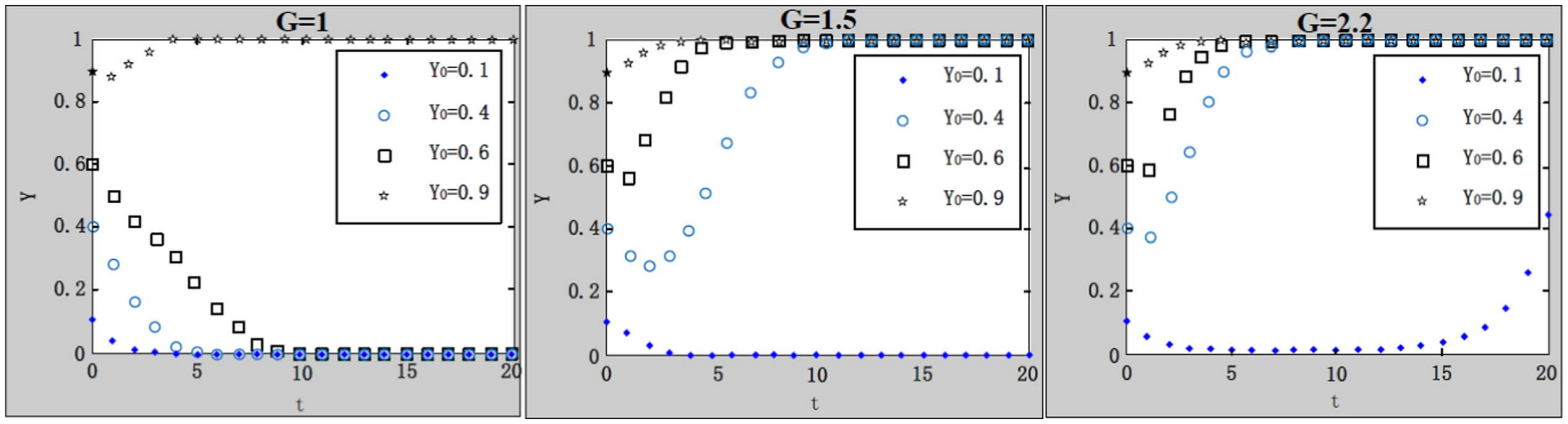
Influence of central government’s penalties on local government’s loose supervision on system evolution.

We take $\begin{array}{l} x_{0}=0.4;u=1;R_{1}=4.9;R_{2}=1;A=1;F=4;\\ C_{1}=4;L=5;C_{0}=8.3;\theta_{1}=0.1;\theta_{2}=0.9 \end{array}$ . According to the parameter settings, the values of G=1;1.5;2.2 are selected, respectively, and the results are shown in Figure 9.

Because it’s a bit of a dilemma in which local governments are responsible for both policies implementation from central government and regional economy development, local government’s work safety supervision on enterprises may deviate from the central government’s goal. So it is essential that the central government inspect local government’s supervision on enterprises. With the increase of the central government’s penalties on local governments’ loose supervision, the system gradually evolves to the desired evolutionary stable point. It is indicated that the central government’s inspection on local governments can effectively urge local governments to perform strict supervision, thus indirectly encouraging enterprises to purchase work safety service.

(7) Influence of rewards from the central government to local governments for strict supervision on the evolution result.

**Figure 10:**
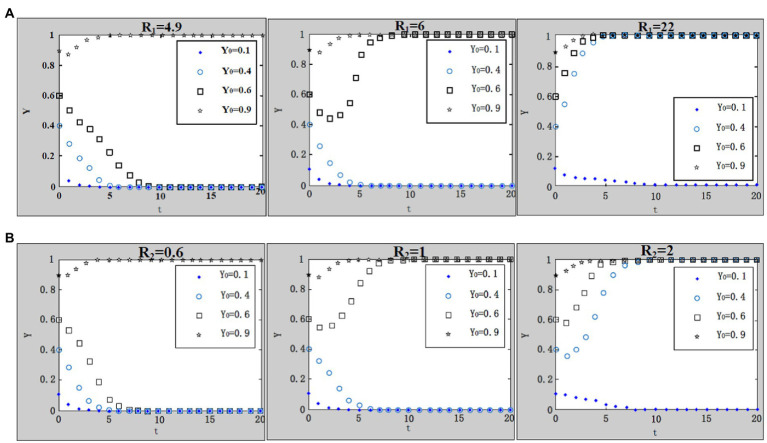
**(A)** Influence of rewards ( $R_{1}$ ) from the central government to local governments for strict supervision on the evolution result. **(B)** Influence of rewards ( $R_{2}$ ) from the central government to local governments for strict supervision on the evolution result.

We take $\begin{array}{l} x_{0}=0.4;u=1;G=1.1;A=1;F=4;C_{1}=4;\\ L=5;C_{0}=8.3;\theta_{1}=0.1;\theta_{2}=0.9 \end{array}$ . According to the parameter settings, the values of $R_{1}=4.9;6;22$ and $R_{2}=0.6;1;2$ are selected, respectively, and the results are shown in Figures 10A,B. There usually will be rewards, such as subsidies and official promotion, from the central government to local governments as long as local governments supervise enterprises strictly, regardless of the enterprises’ strategies on purchase of work safety service. Due to the specific work safety conditions in enterprises, rewards from the central government to local governments vary. It is not difficult to discover that with the increase of rewards, the system in both Figures 10A,B evolves to the desired evolutionary stable point. Logically, when enterprises choose to purchase work safety service, the work safety conditions of the whole administrative region should be considerably improved with work safety accidents declining, hence local governments will obtain more rewards from the central government, then system converges to the desired evolutionary stable point much faster; on the contrary, when enterprises choose not to purchase work safety service, system converges to the desired evolutionary stable point relatively slow, even with the rewards from the central government to local governments.

## Conclusion and discussion

### Research conclusion

Work-safety-service demands always come first in the formation and development of the work-safety-service market. How governments formulate and implement safety-related policies, encourage enterprises to externalize their internal safety management needs into the market and urge enterprises to purchase work safety service are solid prerequisites and foundations of work-safety-service market. Therefore, we create an evolutionary game scenario involving enterprises and local governments under a subsidy and punishment mechanism from the central government. Several conclusions, which are obtained through the analysis of replicator dynamics and stability of equilibrium points, provide theoretical reference for policy formulation and implementation and cultivation of work-safety-service market.

According to the assumptions and parameter settings in the model, the stability of each equilibrium point is discussed in the following three cases (12 specific situations in total).

(1) When $\begin{array}{l} \left(\theta_{2}-\theta_{1}\right)\left(F+L\right)+A+u-C_{0}>\\ \left(\theta_{2}-\theta_{1}\right)\left(F+L\right)-C_{0}>0 \end{array}$ , the payoff with work-safety-service purchase for enterprises is dominantly higher than the payoff with work-safety-service non-purchase, no matter what strategy local governments choose. Hence enterprises always choose to purchase work-safety service. Under this scenario, there are four equilibrium points, and the system evolves to the desired evolutionary stable point if and only if the net payoff of local governments with “strict supervision” is dominantly higher than that with “loose supervision.”
(2) When $\left(\theta_{2}-\theta_{1}\right)\left(F+L\right)-C_{0}<\left(\theta_{2}-\theta_{1}\right)\left(F+L\right)+A+u-C_{0}<0$ , the payoff with work-safety-service purchase for enterprises is dominantly lower than the payoff with work-safety-service non-purchase, no matter what strategy local governments choose. Hence enterprises always choose not to purchase work-safety service. Under this scenario, there are four equilibrium points, and the system evolves to the undesired evolutionary stable point if and only if the net payoff of local governments with “strict supervision” is dominantly lower than that with “loose supervision.”
(3) When $\left(\theta_{2}-\theta_{1}\right)\left(F+L\right)-C_{0}<0<\left(\theta_{2}-\theta_{1}\right)\left(F+L\right)+A+u-C_{0}$ , $\theta_{2}G+R_{2}+u-C_{1}<0$ and $\theta_{1}G+R_{1}-A-C_{1}>0$ , there are five equilibrium points, among which (strict supervision, purchase of service; V(1,1) ) and (loose supervision, nonpurchase of service; O(0,0) ) are two evolutionary stable points. The former is a desired evolutionary stable point, while the latter is the otherwise. It is feasible to make the system converge to the desired evolutionary stable point through modifying relative parameters, avoiding the undesired evolutionary stable point. It is demonstrated in the simulation that the system is more likely to converge to the desired evolutionary stable point with the higher initial ratio of local governments choosing “strict supervision”; the system is more likely to converge to the desired evolutionary stable point with the stricter inspection on local governments by the central government, and the stricter ex ante and ex post safety supervision on enterprises by local governments; subsidies from local governments for the purchase of work safety service barely affect the evolutionary trend of the system. The system is more likely to converge to the undesired evolutionary stable point when the ex ante supervision costs of local governments get higher.

### Policy implications

According to the analysis above, when $\left(\theta_{2}-\theta_{1}\right)\left(F+L\right)-C_{0}<0<\left(\theta_{2}-\theta_{1}\right)\left(F+L\right)+A+u-C_{0}$ , $\theta_{2}G+R_{2}+u-C_{1}<0$ and $\theta_{1}G+R_{1}-A-C_{1}>0$ , the system can evolve to the desired evolutionary stable point by modifying relevant parameters. Therefore, it is suggested that:

(1) The central government should put more effort in the inspection of local governments to avoid “loose supervision” or even collusion with local enterprises due to local protection and administrative evaluation. By increasing the rewards of “strict supervision” and penalties of “loose supervision” of local governments, local governments can be urged to strictly supervise local enterprises, thus indirectly encouraging enterprises to purchase work safety service.
(2) The local government should regularly inspect and randomly check the enterprises to help them eliminate potential risks. Appropriate economic subsidies, tax incentives and financing support should also be provided to enable them purchase work safety service and increase their desire to purchase safety service. For enterprises with work safety accidents, the government should investigate the causes, identify the responsibilities and impose severe punishments. Meanwhile, it is of great necessity to improve the ex ante and ex post supervision mechanisms, take up supervision duty, and implement relevant work safety policies.

### Research limitations and future prospects

This paper has some limitations and the following aspects are worth discussing in future research. First, we assume that the strategies of both game players are discrete (i.e., local governments either supervise enterprises strictly or loosely and enterprises either purchase work safety service or not), which means strategy space is discrete and both local governments and enterprises have pure strategies. In each single game among repeated games, every participant could choose one strategy from strategy space. However, in real life, game players might choose a strategy in-between with a probability from strategy space. The results of our study will then be more practically reasonable if studied in continues strategy space. Second, we do not consider the influence of work-safety-service organizations on enterprises. It would be interesting to make work-safety-service organizations a participant whose strategies could be providing work-safety-service with high standard or with low standard. We could then build a tripartite model to investigate evolutionary path of every participant to better foster work-safety-service market and improve work safety conditions in enterprises with appropriate supervision from governments at all levels.

## Data availability statement

The original contributions presented in the study are included in the article/supplementary material, further inquiries can be directed to the corresponding author.

## Author contributions

ZJ contributed to conception and design of the study and wrote the first draft of the manuscript. LW performed the numerical simulation. All authors contributed to the article and approved the submitted version.

## Funding

This work is financially supported by Ministry of Education of People’s Republic of China (grant number 19YJCZH269). This work is also financially supported by the National Natural Science Foundation of China (grant numbers 72004081 and 72074099).

## Conflict of interest

The authors declare that the research was conducted in the absence of any commercial or financial relationships that could be construed as a potential conflict of interest.

## Publisher’s note

All claims expressed in this article are solely those of the authors and do not necessarily represent those of their affiliated organizations, or those of the publisher, the editors and the reviewers. Any product that may be evaluated in this article, or claim that may be made by its manufacturer, is not guaranteed or endorsed by the publisher.
